# Association between Single Nucleotide Polymorphisms in *ERCC4* and Risk of Squamous Cell Carcinoma of the Head and Neck

**DOI:** 10.1371/journal.pone.0041853

**Published:** 2012-07-27

**Authors:** Hongping Yu, Zhensheng Liu, Yu-Jing Huang, Ming Yin, Li-E Wang, Qingyi Wei

**Affiliations:** 1 Department of Epidemiology and Biostatistics, Guiling Medical University School of Public Health, Guilin, China; 2 Departments of Epidemiology, The University of Texas M.D. Anderson Cancer Center, Houston, Texas, United States of America; The Chinese University of Hong Kong, Hong Kong

## Abstract

**Background:**

Excision repair cross-complementation group 4 gene (*ERCC4/XPF*) plays an important role in nucleotide excision repair and participates in removal of DNA interstrand cross-links and DNA double-strand breaks. Single nucleotide polymorphisms (SNPs) in *ERCC4* may impact repair capacity and affect cancer susceptibility.

**Methodology/Principal Findings:**

In this case-control study, we evaluated associations of four selected potentially functional SNPs in *ERCC4* with risk of squamous cell carcinoma of the head and neck (SCCHN) in 1,040 non-Hispanic white patients with SCCHN and 1,046 cancer-free matched controls. We found that the variant GG genotype of rs2276466 was significantly associated with a decreased risk of SCCHN (OR = 0.69, 95% CI 0.50–0.96), and that the variant TT genotype of rs3136038 showed a borderline significant decreased risk with SCCHN (OR = 0.76, 95% CI: 0.58–1.01) in the recessive model. Such protective effects were more evident in oropharyngeal cancer (OR = 0.61, 95% CI: 0.40–0.92 for rs2276466; OR = 0.69, 95% CI: 0.48–0.98 for rs3136038). No significant associations were found for the other two SNPs (rs1800067 and rs1799798). In addition, individuals with the rs2276466 GG or with the rs3136038 TT genotypes had higher levels of *ERCC4* mRNA expression than those with the corresponding wild-type genotypes in 90 Epstein-Barr virus-transformed lymphoblastoid cell lines derived from Caucasians.

**Conclusions:**

These results suggest that these two SNPs (rs2276466 and rs3136038) in *ERCC4* may be functional and contribute to SCCHN susceptibility. However, our findings need to be replicated in further large epidemiological and functional studies.

## Introduction

DNA repair plays a critical role in protecting the genome from insults caused by carcinogenic agents, such as carcinogens presented in tobacco smoke, ultraviolet light and ionizing radiation. Until now, at least four major DNA repair pathways operate in mammals, including nucleotide excision repair (NER), base excision repair (BER), double-strand break repair (DSBR) and mismatch repair (MMR) [1]. NER is one of the most important DNA repair pathways, which removes a variety of bulky lesions, such as chemically induced bulky adducts, ultraviolet light (UV)-induced photodimers, and oxidized bases [2], [3]. DNA repair capacity (DRC) is substantially variable within the general population, and inherited deficiencies in repair capacity may increase an individual’s susceptibility to cancers [4], [5]. Indeed, a number of studies have reported that reduced DRC is associated with an increased risk of several types of human cancer, including those of the head and neck [6], lung [7], breast [8] and skin [9].

The excision repair cross-complementation group 4 gene (*ERCC4*, also known as *XPF*) encodes the ERCC4 protein, a key enzyme in the NER pathway, that forms a tight complex with ERCC1 to function as a structure-specific endonuclease responsible for the 5-primer incision during DNA excision repair [10]. In addition to NER, the ERCC4/ERCC1 complex is suggested to play a role in removal of DNA interstrand cross-links (ICL), DNA double-strand breaks (DSB), and immunoglobulin class switch recombination (CSR) [11], [12], [13], [14] Germ-line mutations in *ERCC4*, among other XP genes in the NER pathway, are associated with some rare inherited human syndromes, such as Xeroderma pigmentosum (XP), Cockayne syndrome (CS) and Trichothiodystrophy (TTD) [15]. These syndromes fit a recessive genetic model, in which heterozygotes are unaffected, but mutant homozygotes manifest the disease [16]. XP patients with defective DNA repair are extremely photosensitive and have a dramatically increased risk for developing skin cancers [17], [18]. XPF-deficient mice were extremely sensitive to ultraviolet irradiation and that these animals showed a severe postnatal growth defect with early death [13]. Chinese hamster cell lines defective in *ERCC4* were found to be hypersensitive not only to UV but also to DNA interstrand cross-linking agents [19], [20]. Evidence has revealed that expression levels of ERCC4 are correlated with risk, progression, response to cisplatin chemotherapy, and clinical outcome of multiple human cancers including head and neck cancer [21], [22], [23], [24], [25], [26], suggesting that altered ERCC4 expression may lead to altered DRC, thereby modulating cancer susceptibility. We previously reported that the relative protein expression level of ERCC4 was significantly lower in the SCCHN cases than in the controls, and the risk of SCCHN associated with low expression of ERCC4 was higher by 11-fold [25]. Vaezi et al. used quantitative immunohistochemistry to measure ERCC4 expression in tumors from a cohort of 80 patients with newly diagnosed SCCHN treated with radiation therapy with or without platinum-based chemotherapy, and they found that high ERCC4 expression correlated with early time to progression of SCCHN, suggesting that ERCC4 expression levels predict clinical response to DNA damaging agents in SCCHN [26].

*ERCC4* is located on chromosome 16p13.12, contains 11 exons, and spans approximately 28.2 kb. To date, a total of 580 single nucleotide polymorphisms (SNPs) in human *ERCC4* have been reported according to the dbSNP database (http://www.ncbi.nlm.nih.gov/projects/SNP/snp_ref.cgi?chooseRs=all&go=Go&locusId=2072). Several studies have investigated the associations between the *ERCC4* polymorphisms and risk of cancers, including cancer of the breast, lung, head and neck, skin, pancreas and bladder cancer, but the results are not consistent [27], [28], [29], [30], [31], [32], [33] [27]–[33]. Considering the important function of the ERCC4 protein in DNA repair, we conducted a hospital-based case-control study with 1040 SCCHN patients and 1046 controls in a non-Hispanic white population to test the hypothesis that common [minor allele frequency (MAF) ≥5%), potentially functional SNPs in *ERCC4* may contribute to the risk of SCCHN. To the best of our knowledge, there is no published candidate SNP study with such a large number of SCCHN cases and controls to evaluate associations between potentially functional SNPs of *ERCC4* and risk of SCCHN.

## Materials and Methods

### SCCHN Patients and Cancer-free Controls

The recruitment of study subjects for the present study has been previously described [34]. Briefly, 1040 newly diagnosed, untreated patients with histologically confirmed SCCHN and 1046 cancer-free controls were recruited from The University of Texas M.D. Anderson Cancer Center between October 1999 and October 2007. Patients with second primary tumors, primary tumors of the skin, nasopharynx, sinonasal tract, and/or any histopathologic diagnose other than squamous cell carcinoma were excluded. The cancer-free controls were recruited from genetically unrelated individuals who were not seeking health care, but accompanying the patients to visit The M. D. Anderson Cancer Center clinics. We frequency matched the controls to the SCCHN patients by age (±5 years) and sex. Only non-Hispanic white patients and cancer-free controls were included in this analysis, because genotype frequencies can vary between ethnic groups and few patients of ethnic minority groups were recruited. The purpose of frequency matching was to control confounding in the interest of the main effect of the *ERCC4* polymorphisms. All potential study subjects have signed a written informed consent and then were interviewed to gather demographic data and history of tobacco and alcohol use. Among all eligible subjects, the response rate for SCCHN patients and cancer-free controls were approximately 93% and 85%, respectively. Subjects who had smoked more than 100 cigarettes in their lifetimes were defined as ‘ever smokers’. Ever smokers who had quit smoking more than 1 year previously were defined as former smokers and the others as current smokers. Subjects who had drunk alcoholic beverages at least once a week for more than 1 year previously were defined as ‘ever drinkers’. Ever drinkers who had quit drinking more than 1 year previously were defined as former drinkers and the others as current drinkers. Each subject donated one-time 30-mL of blood, which was mainly for DNA repair phenotype assay [35] and 1 ml fresh blood was centrifuged to separate plasma and Buffy coat to be stored in freezer for genomic DNA extraction with a DNA blood Mini Kit (Qiagen, Valencia, CA) according to the manufacturer’s instructions. The research protocol was approved by the M.D. Anderson Institutional Review Board.

### SNP Selection and Genotyping

To determine the association between common potentially functional *ERCC4* SNPs and SCCHN risk, we first used a computational tool of SNP functional prediction (http://snpinfo.niehs.nih.gov/snpfunc.htm) from those 508 SNPs, which were reported in the dbSNP database, to select any SNPs with any predicted functionality: (1) affecting transcription factor binding sites (TFBS) activity in the putative promoter region (*ERCC4* gene with 2-kb upstream from the first exon), (2) affecting the micro RNA (miRNA) binding site activity, (3) the introduction of premature termination codon (PTC), or (4) single amino acid substitutions or changing the frame of the protein coding region. A total of 47 potentially functional *ERCC4* SNPs were screened out. After we limited the SNPs to those with MAF ≥5% in the HapMap CEU (Utah residents with Northern and Western European ancestry from the CEPH collection) population, seven potentially functional SNPs were identified, which were rs3136038, rs6498486, rs1799797, rs1800067, rs3743538, rs2276465, and rs2276466. We found that rs6498486, rs1799797, rs3743538, rs2276465, and rs2276466 were in strong linkage diseqilibrium (LD) with each other (r^2^>0.83) in the HapMap CEU population. Thus, we selected rs2276466, and other three SNPs (i.e., rs3136038, rs1799798, and rs1800067) for further genotyping. Among these four SNPs, rs3136038 and in rs1799798 were predicted in an ELF-1 binding site and a H4TF2-binding site of *ERCC4* gene, respectively, and rs2276466 was predicted in a putative miRNA binding site for hsa-miR-877 located in in the 3′ untranslated region (3′UTR) of *ERCC4*. rs1800067 (Arg415Gln) is a non-synonymous coding SNP.

Genotyping data of the rs1799798 and rs1800067 polymorphisms were available from our SNPlex database. These two SNPs were genotyped by using the SNPlex assay in the DNA Core Facility at M.D. Anderson Cancer Center, according to the protocol of manufacturer (Applied Biosystems, Foster City, CA). The data output from the SNPlex assay was analyzed with the GeneMapper software (Applied Biosystems) to determine the genotypes. The samples failed to be genotyped in the SNPlex assay were re-evaluated with the polymerase chain reaction (PCR)-restriction fragment length polymorphism (RFLP) assay. Approximately 10% of the samples were randomly selected and repeated with the PCR-RFLP assay, and the results were 100% concordant. The other two SNPs, i.e., rs2276466 and rs3136038, were unavailable from the SNPlex database and were genotyped by using the TaqMan assay (Applied Biosystems, Foster City, CA) according to the manufacturre’s recommendations. The PCR amplification was run, and the plate was read using a TaqMan 7900 HT sequence detection system (Applied Biosystems, Foster City, CA). The analyzed fluorescence results were then auto-called into the genotypes using the built-in SDS2.3 software of the system.

### Correlation between Polymorphism Genotype and Gene Expression Levels

The *ERCC4* mRNA expression data by the genotypes of *ERCC4* polymorphisms are publicly available online (http://app3.titan.uio.no/biotools/help.php?app=snpexp) [36]. The genotyping data were derived from the HapMap Phase II release 23 data set consisting of 3.96 million SNP genotypes from 90 HapMap CEU [37]. The mRNA expression data for *ERCC4* in Epstein-Barr virus (EBV)-transformed lymphoblastoid cell lines were derived from the same 90 individuals [38]. Student’s *t* test was used to compare the differences in mRNA expression levels among genotype groups, and the linear trend of mRNA expression levels among genotypes was tested using linear regression models.

### Statistical Analysis

Chi-square test was used to evaluate differences in frequency distributions of demographic characteristics, selected variables (smoking status and alcohol use), and allele frequencies of the four *ERCC4* SNPs between cases and controls. The deviation from the Hardy-Weinberg equilibrium (HWE) among controls for each SNP was tested by a Chi-square goodness-of-fit χ2 test. Genotype frequencies were compared using the Cochran Armitage trend test. Haplotype frequencies and individual haplotypes were generated using SAS PROC HAPLOTYPE. To assess the association between ERCC4 genotypes/haplotypes and disease status, the crude and adjusted odds ratio (OR) and 95% confidence interval (CI) were estimated using unconditional univariate and multivariate logistic regression models with and without adjustment for age, sex, smoking status, and alcohol use. Additional stratified analyses of associations of *ERCC4* genotypes with SCCHN risk by subgroups of age, sex, smoking and drinking status and tumor sites were also performed, followed by analyses of gene–environment interactions, which were evaluated by the *P* value for the interaction term in multivariate logistic regression models with adjustment for age, sex, smoking and drinking status. To determine whether the main effect of the *ERCC4* SNPs was independent of other known risk factors, the selected variables such as age, sex, smoking/drinking status were included in the multivariate logistic regression analyses. Two models were fitted. The first model included the selected variables. The second model included all the selected variables and the four polymorphisms of interest, the aim being to further assess the independent effects of the polymorphisms. Receiver operating characteristic curves were used to summary statistics of the area under the curve (AUC) were calculated for all logistic regression models using the SAS statistical software program. All tests were two-sided with a significance of P<0.05. All statistical analyses were performed by using the SAS software (version 9.1.3; SAS Institute Inc., Cary, NC).

## Results

### Characteristics of the Study Population

We examined associations between the selected *ERCC4* SNPs and risk of SCCHN in 1040 cases and 1046 controls of non-Hispanic whites. Among all SCCHN cases, 307 (29.5%) had primary tumors of the oral cavity, 531 (51.0%) of the oropharynx and 202 (19.4%) of the hypopharynx/larynx. The call rate in each SNP genotyping was 100% for rs2276466, rs1800067 and rs3136038, respectively, and 99.8% for rs1799798. The distributions of selected characteristics of the SCCHN patients and controls are shown in **Table 1** **.** The mean age was 57.0 years (±11.2 years, range, 18–90 years) for cases and 56.6 years (±11.0 years, range, 20–87 years) for controls. Cases and controls were appeared to be adequately matched by age and sex (*P* = 0.655 and 0.430, respectively). However, compared with the controls, the cases were more likely to be smokers and drinkers (*P*<0.001 for both). We found that smoking and alcohol drinking was associated with a significant increased risk of SCCHN, respectively (OR = 2.18; 95% CI: 1.81–2.64 for smoking; OR = 1.71; 95% CI: 1.41–2.18 for drinking). Furthermore, interaction between smoking and alcohol drinking was also significant (*P* for interaction  = 0.001), and the risk was greater among people who smoked and drank (OR = 3.34; 95% CI: 2.63–4.30) compared to those who neither smoked nor drank.

**Table 1:** Demographic characteristics of SCCHN cases and cancer-free controls.

Variables	Cases	Controls	*P*-value†
	No. (%)	No. (%)	
All subjects	1040 (100%)	1046 (100%)	
Age (years)			0.655
≤ 57‡	559 (53.8)	552(52.8)	
>57	481 (47.2)	494 (47.2)	
Sex			0.430
Male	780 (75.0)	800 (76.5)	
Female	260 (25.0)	246 (23.5)	
Smoking Status			<0.0001
Never	293 (28.2)	512 (49.0)	
Former smoker	353 (33.9)	380 (36.3)	
Current smoker	394 (37.9)	154 (14.7)	
Alcohol use			<0.0001
Never	285 (27.4)	449 (42.9)	
Former	226 (21.7)	172 (16.4)	
Current	529 (50.9)	425 (40.6)	
Tumor site			
Oral cavity	307 (29.5)	–	
Oropharynx	531 (51.0)	–	
Hypopharynx/Larynx§	202 (19.4)	–	

† Two-sided Chi-square test.

‡ The median age of the controls.

§ Including 40 hypopharyngeal cancer cases and 162 laryngeal cancer cases.

### Association between *ERCC4* Polymorphisms and SCCHN Risk

**Figure 1:**
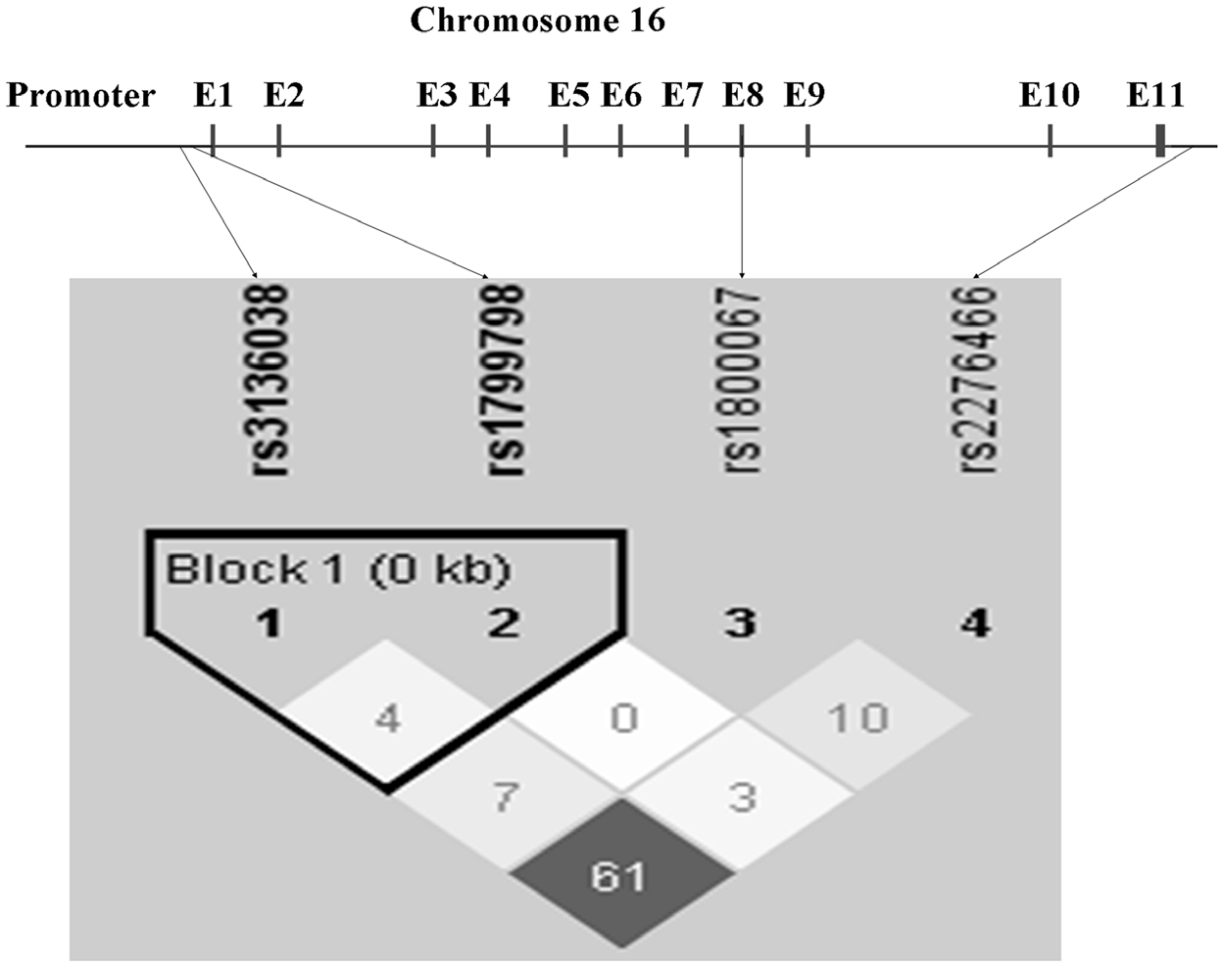
*ERCC4* gene structure, locations of the four potentially functional single nucleotide polymorphisms (SNP), and linkage disequilibrium (LD) plot for these SNPs. The color of each square represents the pairwise *r* ^2^; the darker, the stronger *r* ^2^, with dark black representing *r* ^2^ = 1 and pure white representing *r* ^2^ = 0.

The *ERCC4* gene structure and locations of the four SNPs (rs2276466, rs3136038, rs1799798, and rs1800067) are displayed in **Figure 1** **.** Among the four SNPs, rs3136038 and rs1799798 are located at the predicted transcription factor binding site of the 5′ untranslated region (UTR) of *ERCC4*, rs2276466 is located at the predicted miRNA-binding site of the 3′UTR of *ERCC4*, and rs1800067 is located at exon 8 in *ERCC4*. LD analysis revealed that these four SNPs were not in LD among the controls (**Figure 1**). **Table 2** shows the allele and genotype frequencies of the four *ERCC4* SNPs for cases and controls. The observed genotype distributions for these four SNPs were all in agreement with HWE in the control subjects (*P* = 0.143 for rs3136038, *P* = 0.830 for rs1799798, *P* = 0.187 for rs1800067, *P* = 0.064 for rs2276466). The allele frequencies and genotype frequencies of these SNPs under an additive model did not differ between cases and controls (**Table 2**).

**Table 2:** Allele frequencies, genotype frequencies, and OR and 95% CI for association between *ERCC4* polymorphisms and SCCHN risk.

Genotype	Cases (No. = 1040)		Controls (No. = 1046)		*P*-Value	Crude OR (95%CI)	Adjusted OR^c^ (95%)
	No.	%	No.	%			
rs2276466							
CC	522	50.2	529	50.6	0.387^a^	Reference	Reference
CG	443	42.6	412	39.4		1.09 (0.91–1.31)	1.13 (0.93–1.36)
GG	75	7.2	105	10.0		0.72 (0.53–1.00)	0.73 (0.52–1.02)
G allele frequency		28.5		29.7	0.385^b^		
CC/CG	965	92.8	941	90.0	0.022^b^	Reference	Reference
GG	75	7.2	105	10.0		0.70 (0.51–0.95)	0.69 (0.50–0.96)
rs1800067							
GG	837	80.5	829	79.2	0.512^a^	Reference	Reference
AG	195	18.7	209	20.0		0.92 (0.74–1.15)	0.92 (0.73–1.16)
AA	8	0.8	8	0.8		0.99 (0.37–2.65)	1.38 (0.50–3.79)
A allele frequency		10.1		10.8	0.519^b^		
GG/AG	1032	99.2	1038	99.2	0.991^b^	Reference	Reference
AA	8	0.8	8	0.8		1.01 (0.38–2.69)	1.40 (0.51–3.85)
rs1799798^d^							
GG	846	81.4	861	82.6	0.501^a^		Reference
AG	185	17.8	173	16.6		1.09 (0.87–1.37)	1.10 (0.87–1.40)
AA	8	0.8	8	0.8		1.02 (0.38–2.73)	0.93 (0.33–2.57)
A allele frequency		9.7		9.1	0.504^b^		
GG/AG	1031	99.2	1034	99.2	0.995^b^	Reference	Reference
AA	8	0.8	8	0.8		1.01 (0.38–2.69)	0.90 (0.33–2.52)
rs3136038							
CC	452	43.5	458	43.8	0.379^a^	Reference	Reference
CT	483	46.4	452	43.2		1.09 (0.91–1.31)	1.10 (0.91–1.33)
TT	105	10.1	136	13.0		0.72 (0.53–1.00)	0.80 (0.59–1.08)
T allele frequency		33.3		34.6	0.379^b^		
CC/CT	935	89.8	910	87.0	0.038^b^	Reference	Reference
TT	105	10.1	136	13.0		0.70 (0.51–0.95)	0.76 (0.58–1.01)

a Cochran-Armitage trend test for genotype distributions under an additive genetic model between cases and controls.

b Chi square test for allele and genotype distributions under a recessive genetic model between cases and controls.

c Adjusted by age, gender, smoking status and alcohol status in logistic regression models.

d One case and four controls genotyping were failed for rs1799798.

We tested the hypothesis that the variant homozygous genotypes of these *ERCC4* SNPs are associated with risk of SCCHN, assuming a recessive genetic model (i.e., only the variant homozygous genotype was considered as the risk genotype) as for XP patients. As shown in **Table 2**, significant differences in the genotype distributions under a recessive genetic model were found between the cases and controls for rs2276446 and rs3136038 (*P* = 0.022 for rs2276466; *P* = 0.038 for rs3136038) but not for rs1800067and rs1799798 (*P* = 0.995 for rs1800067; *P* = 0.991 for rs1799798). The rs2276466 GG genotype was associated with a significantly decreased risk of SCCHN (GG vs. CC/CG: adjusted OR = 0.69; 95% CI, 0.50–0.96) with adjustment for age, sex, smoking, and alcohol use. The rs3136038 TT genotype showed a borderline significant association with a decreased risk of SCCHN (TT vs. CC/CT: adjusted OR = 0.76; 95% CI: 0.58–1.01). In the stratified analysis by tumor sites, we found that both the rs2276466 GG and rs3136038 TT genotypes were statistically significantly associated with a decreased risk of oropharyngeal cancer (GG vs. CC/CG: adjusted OR = 0.61; 95% CI, 0.40–0.92 for rs2276466; TT vs. CC/CT: adjusted OR = 0.69; 95% CI: 0.48–0.98 for rs3136038) but not for other tumor sites. No associations were observed between the genotypes of other two SNPs (rs1799798 and rs1800067) and risk of SCCHN either overall or by sub sites (**Table 2**).

We further assessed the association between haplotypes of these four independent SNPs and risk of SCCHN (**Table 3**). We inferred twelve haplotypes of *ERCC4* polymorphisms, five of which had frequency greater than 0.05. Using the most common haplotype “CCGG” as the reference, we found that all other four common haplotypes were not significantly associated with risk of SCCHN. Seven haplotypes had frequencies less than 0.05, so we combined them into a single “minor haplotype” category, which was associated with a reduced risk of SCCHN (adjusted OR = 0.57; 95% CI: 0.41–0.81). Because the sample size for each minor haplotype was very small, we had limited statistical power to detect which one was significantly associated with the risk of SCCHN.

**Table 3:** Association between *ERCC4* haplotypes and risk of SCCHN.

Haplotype	Cases (No. = 2078)		Controls (No. = 2084)		Crude OR (95% CI)	Adjusted OR (95% CI)^a^
	No.	%	No.	%		
C-C-GG	1137	54.72	1102	52.88	Reference	Reference
G-T-G-G	378	18.19	400	19.19	0.92 (0.78–1.08)	0.92 (0.78–1.09)
C-C-A-G	193	9.29	170	8.16	1.10 (0.88–1.37)	1.11 (0.88–1.39)
G-T-G-A	182	8.76	172	8.25	1.03 (0.82–1.28)	1.07 (0.85–1.36)
C-T-G-G	126	6.06	139	6.07	0.88 (0.68–1.13)	0.88 (0.67–1.15)
All minor haplotypes	62	2.98	101	4.85	0.60 (0.43–0.83)	0.57 (0.41–0.80)

a Adjusted by age, gender, smoking status and alcohol status in logistic regression models. The Chi-square test for haplotype frequency distribution in the cases and controls was 12.87 with five degrees of freedom (*P* = 0.025).

Including seven haplotypes, each with a frequency less than 0.05.

Further stratified analysis by sex, age, smoking and drinking statuses showed that the decreased risk of SCCHN associated with the rs2276466 GG genotype was more evident among older subjects, non-smokers, and drinkers. Such a protective effect of the rs3136038 TT genotype was also more evident among non-smokers. But interactions were not found between selected variables and these two SNPs (rs2276466 and rs3136038), suggesting that no risk effects were modified by these variables under investigation (**Table 4**). No associations were observed between the genotypes of other two SNPs (rs1799798 and rs1800067) and the risk of SCCHN in the stratification analysis by these selected covariates (**data not shown**). In the stratified analysis by tumor sites, we found that both the rs2276466 GG and rs3136038 TT genotypes were statistically significantly associated with a decreased risk of oropharyngeal cancer (GG vs. CC/CG: adjusted OR = 0.61; 95% CI, 0.40–0.92 for rs2276466; TT vs. CC/CT: adjusted OR = 0.69; 95% CI: 0.48–0.98 for rs3136038) but not for other tumor sites. No associations were observed between the genotypes of other two SNPs (rs1799798 and rs1800067) and risk of SCCHN either overall or subsites of SCCHN (**Table 4**).

**Table 4:** Stratified analysis for SCCHN risk associated with genotypes of *ERCC4* rs3136038 *and* rs2276466.

Variables	rs3136038				rs2276466			
	Cases/Controls		Crude OR (95% CI)	Adjusted OR (95% CI)^a^	Cases/Controls		Crude OR (95% CI)^†^	Adjusted OR (95% CI)^a^
	CC/CT	TT			CC/CG	GG		
All subjects	935/910	105/136	0.70 (0.51–0.95)	0.76 (0.58–1.01)	965/941	75/105	0.70 (0.51–0.95)	0.69 (0.50–0.96)
Age (years)								
≤57^b^	502/483	57/69	0.80 (0.55–1.15)	0.80 (0.54–1.17)	516/499	43/53	0.80 (0.51–1.23)	0.80 (0.51–1.23)
>57	433/427	48/67	0.71 (0.48–1.05)	0.75 (0.49–1.14)	449/442	32/52	0.61 (0.38–0.96)	0.58 (0.36–0.95)
			*P* for interaction 0.807				*P* for interaction = 0.395	
Sex								
Male	702/701	78/99	0.79 (0.57–1.08)	0.81 (0.58–1.12)	722/719	58/81	0.71 (0.50–1.02)	0.74 (0.51–1.06)
Female	233/209	27/37	0.66 (0.39–1.12)	0.66 (0.38–1.18)	243/222	17/24	0.65 (0.34–1.24)	0.56 (0.28–1.12)
			*P* for interaction = 0.499				*P* for interaction = 0.500	
Smoking Status								
No	268/436	25/76	0.54 (0.33–0.86)	0.55 (0.34–0.88)	275/459	18/53	0.57 (0.33–0.99)	0.57 (0.33–1.00)
Yes	667/474	80/60	0.95 (0.66–1.35)	0.96 (0.66–1.39)	690/482	57/52	0.77 (0.52–1.14)	0.78 (0.52–1.17)
			*P* for interaction = 0.059				*P* for interaction = 0.392	
Alcohol use								
No	260/407	25/42	0.76 (0.48–122)	0.78 (0.49–1.25)	260/407	25/42	0.93 (0.56–1.57)	0.94 (0.56–1.59)
Yes	705/534	50/63	0.76 (0.54–1.06)	0.77 (0.54–1.10)	705/534	50/63	0.60 (0.41–0.89)	0.60 (0.40–0.91)
			*P* for interaction = 0.929				*P* for interaction = 0.225	
Tumor site								
Oral cavity	269/910	38/136	0.94 (0.62–1.41)	0.94 (0.62–1.41)	280/941	27/105	0.83 (0.52–1.34)	0.83 (0.52–1.34)
Oropharynx	484/910	47/136	0.69 (0.48–0.98)	0.69 (0.48–0.98)	498/941	33/105	0.61 (0.40–0.92)	0.61 (0.40–0.92)
Hypopharynx/Larynx	182/910	20/136	0.76 (0.44–1.33)	0.76 (0.44–1.33)	187/941	15/105	0.71 (0.38–1.33)	0.71 (0.38–1.33)

a Adjusted by age, gender, smoking status and alcohol status in logistic regression models.

b The median age of the controls.

Finally, we fit six separate multivariable logistic regression models to identify independent effects of the *ERCC4* SNPs on SCCHN risk (**Table 5**). We separately added each of SNPs and the combined genotypes of all SNPs to the model that included other covariates, such as age, sex, and smoking/drinking status. The AUC was the greatest for the model that included rs2076466 polymorphism (AUC  = 0.667; *P* = 0.026) and the model that included rs3136038 polymorphism (AUC  = 0.666; *P* = 0.110), which was toward significance.

**Table 5:** Comparison of Logistic Regression Models with and without genotype for 4 SNPs in *ERCC4.*

Model	Df^a^	−2LogL^b^	LRT^c^	Df^d^	*P*-value^e^	AUC
Constant^f^		2891.793				
Constant +4 variables	4	2710.373	181.42	4	<0.001	0.663
Constant +4 variables + rs2076466	6	2703.911	6.462	2	0.040	0.667
Constant +4 variables + rs3136038	6	2705.967	4.406	2	0.110	0.666
Constant+4 variables + rs1799798	6	2709.727	0.646	2	0.724	0.664
Constant +4 variables + rs180067	6	2709.46	0.913	2	0.633	0.664
Constant +4 variables + all SNPs	12	2701.758	8.615	8	0.375	0.668

a Number of variables in the logistic regression model.

b −2Log likelihood value for the logistic regression model.

c Likelihood ratio test for evaluating the fit of the model.

d Difference in the number of variables when comparing two models.

e P value for the likelihood ratio test to compare two models.

f Model with constant only.

g Model with constant and covariates such as age, sex, smoking status and alcohol status.

### Correlation between *ERCC4* Polymorphism Genotype and Expression Levels of *ERCC4*

**Figure 2:**
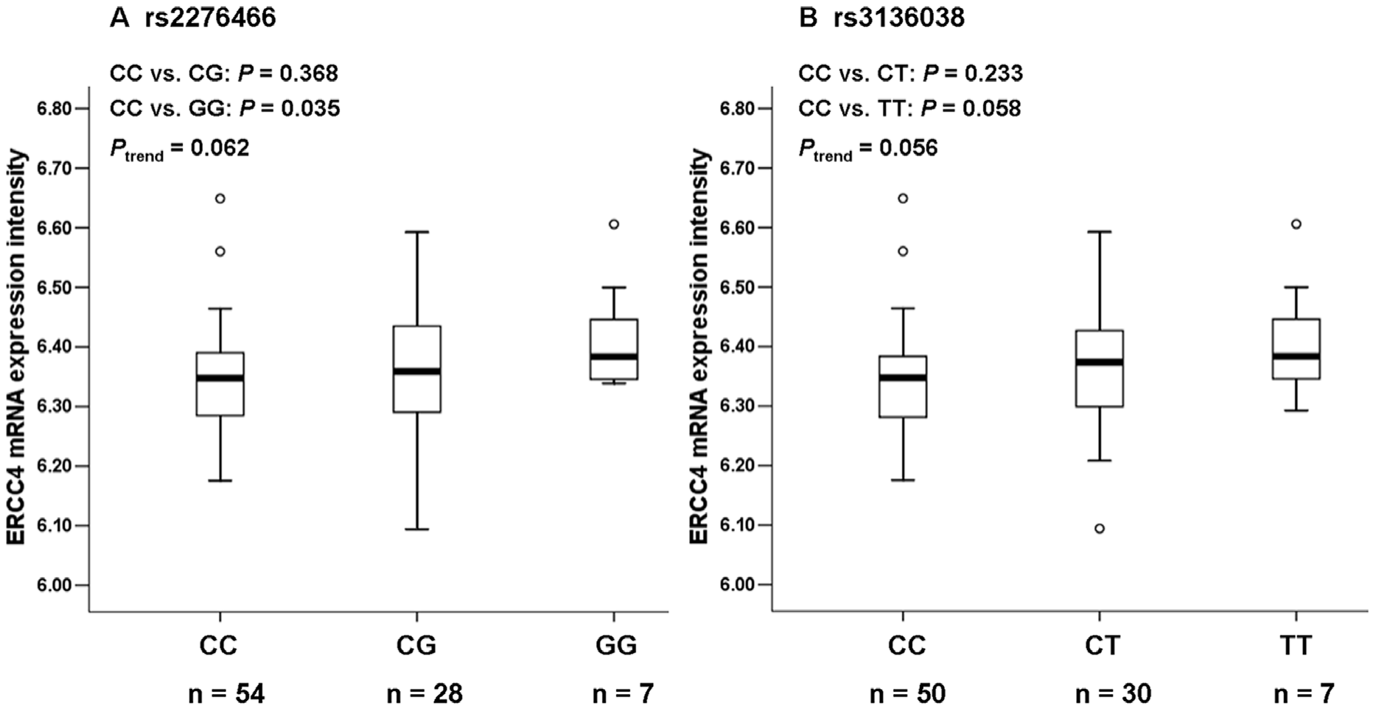
Levels of *ERCC4* mRNA expression by rs2276466 and rs3236038 genotypes in 90 Epstein-Barr virus (EBV)-transformed lymphoblastoid cell lines derived from Caucasians. (A) *ERCC4* expression by the rs2276466 genotypes. (B) *ERCC4* expression by the rs3136038 genotypes. Both the variant GG genotype of rs2276466 and the variant TT genotype had higher levels of *ERCC4* mRNA expression, compared to the corresponding wild-type genotypes (CC vs. GG: *P* = 0.035 for rs2276466; CC vs. TT: *P* = 0.058 for rs3136038). The trend test for the effects of the variants allele of these two SNPs on *ERCC4* expression levels were toward significance (*P*_trend_  = 0.062 for rs2276466; *P*_trend_  = 0.056 for rs3136038). The box represents the interquartile range, which contains 50% of the values. The lower and the upper edges of the box plot are the first quartile (25th percentile) and the third quartile (75th percentile), respectively. The line across the box indicates the median value. The ends of the vertical lines extend to a maximum of 1.5 times the interquartile. In the box plots outliers are marked as dots, which are more than 1.5-fold the box length away from the upper or lower edge of the box. (One and three individuals’ genotyping data were unavailable for rs2276466 and rs3136038, respectively.).

To explore functional relevance of *ERCC4* polymorphisms, we performed correlation analyses between expression levels of *ERCC4* mRNA and genotypes of rs2276466 and rs3136038 using the *ERCC4* mRAN expression data in EBV-transformed lymphoblastoid cell lines derived from 90 HapMap CEU individuals and the genotyping data from the same individuals. We found that the individuals with the GG genotype (n = 7) of rs2276466 had higher levels of *ERCC4* expression of (*P* = 0.035) than those with the rs2276466 CC genotype (n = 54). We also found that individuals with the TT genotype (n = 7) of rs3136038 had higher levels of *ERCC4* expression (*P* = 0.058) than those with the rs3136038 CC genotype (n = 50). Furthermore, the trend test for the effects of the rs2276466 G allele and the rs3136038 T allele on the *ERCC4* expression were toward significance (*P*_trend_  = 0.062 for rs2276466; *P*_trend_  = 0.056 for rs3136038) (**Figure 2**).

## Discussion

In this study, we investigated associations between four selected potentially functional SNPs of *ERCC4* and risk of SCCHN in a non-Hispanic white population. Our data showed that the variant GG genotype of rs2276466 was significantly associated with a decreased risk of SCCHN and that the variant TT genotype of rs3136038 showed a borderline significant decreased risk with SCCHN in recessive models. Such protective effects were more evident in oropharyngeal cancer, older subjects, non-smokers and alcohol drinkers. But no significant interaction effect between any of the variables analyzed was found. No significant associations were observed between the other two SNPs (i.e., rs1799798 and rs1800067) and risk of SCCHN. In addition, the observed significant associations are supported by data that the variant homozygous genotypes of rs2276466 and rs3136038 were associated with increased mRNA expression levels of *ERCC4* in EBV-transformed lymphoblastoid cell lines derived from Caucasians. Our findings suggested that rs2276466 and rs3136038 in *ERCC4* may be functional and contribute to SCCHN susceptibility. Although the published genome-wide association study (GWAS) have not identified *ERCC4* variants as susceptibility loci [39], it was not possible to evaluate the effects of the genotypes for those potentially functional SNPs in *ERCC4* in the GWAS, in which no enough samples nor information about exposures were available for analysis of such gene-environment interactions. In the present study, the data were available for us to evaluate the potential interaction effects between *ERCC4* polymorphisms and selected variables on SCCHN risk, and no significant interaction effects were found, suggesting that no risk effects were modified by these variables under investigation. However, our findings need to be confirmed by large-scale studies.

A few studies have investigated the association between *ERCC4* SNPs and risk of SCCHN, which focused on rs1800067 (Arg415Gln) with inconsistent results. Only three published studies with small sample sizes have examined rs1800067 in association with risk of laryngeal cancer in Polish and German populations, but no significant associations were found in these studies [32], [40], [41], which are similar to our results. No published studies, so far, have reported the association between rs2276466, rs3136038 and rs1799798 and SCCHN risk.

rs2276466 and rs3136038 reside in the predicted miRNA-binding site of the 3′UTR region and the transcription factor binding site of the 5′UTR region of *ERCC4*, respectively. Although functional significance of these two SNPs remains unclear, a growing body of evidence indicated that SNPs located at miRNA-binding sites or transcription factor binding sites are likely to affect gene expression levels by modifying miRNA targeting activity or by altering DNA binding properties of transcription factors and thus may contribute to susceptibility to cancer [42], [43], [44], [45], [46], [47]. Interestingly, we found that the homozygous variant genotypes of both rs2276466 and rs3136038 were indeed associated with increased mRNA expression levels of *ERCC4* in EBV-transformed lymphoblastoid cell lines derived from Caucasians, which supports the observed associations of these two homozygous variant genotypes with a decreased risk of SCCHN. Our previous studies also showed that decreased expression of *ERCC4* mRNA and protein in lymphocytes was associated with an increased risk of SCCHN [48]. Recently, Vaezi et al. reported that low expression of ERCC4 was associated with longer progression-free survival in patients with SCCHN treated with DNA damaging agents [26]. Both *in vitro* and *in vivo* studies from XPF-deficient cells and mice showed that lower XPF expression was associated with an exquisite sensitivity to DNA damaging agents [12], [49], [50]. Taken together, these two potentially functional SNPs (rs2276466 and rs3136038) may alter mRNA expression levels of *ERCC4*, thereby affecting DRC and modulating cancer susceptibility. However, the exact molecular mechanisms underlying the observed effects need further investigation.

In summary, we report that two potentially functional *ERCC4* SNPs, i.e., rs2276466 and rs3136038, may influence mRNA expression levels of *ERCC4* and thus contribute to genetic susceptibility to SCCHN. Nevertheless, some limitations in the current study should be considered. Firstly, since this was a hospital-based case-control study, the issue of selection bias was unavoidable. However, the hypothesis tested in this study was a genotype-driven rather than an environment-driven, and it is unlikely to have improperly selected individuals related to genotype. Secondly, *ERCC4* may play an important role in NER involved in removal of a variety of bulky DNA adducts such as those formed by tobacco carcinogens PAHs. Therefore, potential residual confounding may exist, if only the smoking status but not the smoking dose and duration data were available in our current study. For example, we observed a protective effect of the homozygous variant genotypes of rs2276466 and rs3136038 in non-smokers but not smokers, which is difficult to explain. Hence, detailed information for smoking or drinking habits (dose and duration) should be taken into account in future studies. Thirdly, although the sample size of our study was relatively large, the statistical power was still limited in the analyses of some groups with small sample sizes. For example, in the analyses by tumor sites, the *ERCC4* rs2276466 and rs3136038 were only found to be associated with risk of oropharyngeal cancer but not for cancers of oral cavity, hypopharynx and larynx. In addition, no significant associations were observed between the other two SNPs (i.e., rs1799798 and rs1800067) and risk of SCCHN in the recessive model. This lack of significance could be either because there was no such effect or because the small sample size in these groups (only 307 oral cavity cancer patients and 203 larynx and hypopharynx patients, and only eight cases and eight controls had the homozygous variant genotype for rs1799798 and rs1800067, respectively) limited the statistical power to detect a modest effect on cancer risk. Hence, our findings should be interpreted with caution. Fourthly, due to the multiple comparisons being made, we cannot rule out the possibility of false-positive associations. When correcting our estimates for multiple testing according to Bonferroni, no significant associations were found. Therefore, our findings need to be replicated, and more profound functional studies are necessary to explore the functional relevance of these SNPs and the underlying molecular mechanisms for the observed associations.
